# Microbiological Analysis of Plaque and Its Composition in Three Patient Groups under Different Orthodontic Treatments

**DOI:** 10.3390/dj12060168

**Published:** 2024-06-04

**Authors:** Niccolò Cenzato, Chiara Occhipinti, Elena D’amici, Paolo Savadori, Benedetta Baldini, Cinzia Maspero

**Affiliations:** 1 Department of Biomedical, Surgical and Dental Sciences, School of Dentistry, University of Milan, 20122 Milan, Italychiara.occhipinti@unimi.it (C.O.); elena_damici@libero.it (E.D.); cinzia.maspero@unimi.it (C.M.); 2 Fondazione IRCCS Cà Granda, Ospedale Maggiore Policlinico, 20122 Milan, Italy; 3 Department of Electronics, Information and Bioengineering, Politecnico Di Milano, 20133 Milan, Italy; benedetta.baldini@polimi.it

**Keywords:** orthodontic treatment, microbiota, dental health survey, periodontal health care, orthodontic appliances

## Abstract

Background: This article analyzes differences in microbiological parameters and periodontal health conditions among three patient groups: those undergoing conventional orthodontic treatment with fixed appliances, patients undergoing orthodontic treatment with clear aligners, and a control group receiving no treatment. Materials and Methods: In this study, 60 patients were enrolled. The microbiological analysis employed a qualitative and semi-quantitative methodology of bacterial morphotype analysis. Results: The analyses revealed a significant difference in favor of clear oral and periodontal health aligners. This could be attributed to better bacterial biofilm removal and reduced mechanical stress on the periodontal ligament, factors facilitated by the ease of clear aligner removal. Significant differences (*p*-value < 0.05) were observed for the Full-Mouth Plaque Score, Full-Mouth Bleeding Score, Plaque Index, and periodontal health assessment measurements. Conclusions: Although overall hygiene appears to be improved in patients in the aligners group compared to those treated with conventional orthodontic appliances, there are no statistically significant results regarding plaque composition. Microbiological aspects will be further addressed using more specific techniques in the follow-up of this research.

## 1. Introduction

In recent years, there has been a growing focus on dental aesthetics, leading patients to desire outcomes based on more natural aesthetic parameters, harmonizing with facial anatomy [1,2]. Currently, orthodontics plays an increasingly crucial role in the general population and the dental field, assuming an interdisciplinary role of greater significance. One of the primary goals of orthodontic treatment is to correct malocclusions, traditionally achieved through fixed appliances such as bands, brackets, arches, ligatures, and auxiliaries [3]. Simultaneously, there has been an increased demand for orthodontic treatments with clear appliances as they meet patients’ desire for less visible and more comfortable orthodontic appliances [4,5]. With the rising demand for malocclusion correction, curiosity arises about the physiological and microbial changes that may occur during treatment.

Furthermore, orthodontic therapy impacts oral hygiene, promoting plaque retention as the structures within the oral cavity provide ample surfaces for bacterial anchorage, leading to biofilm and plaque formation. Additionally, there is an increase in gingival inflammation due to both the heightened bacterial load and the mechanical action exerted by the orthodontic appliances themselves. These conditions can also contribute to enamel demineralization, potentially leading to carious lesions. Therefore, understanding the differences in microbiological aspects and oral and periodontal health between treatments is essential [6,7], especially as new medical devices are introduced as increasingly utilized alternatives to more traditional methods. The existing literature provides conflicting results, with various meta-analyses and studies presenting different perspectives [8,9]. One can cite Socransky’s microbiological studies [10,11,12], which indicate the emergence of specific bacterial species during orthodontic treatment. For instance, Aggregatibacter actinomycetemcomitans, primarily found in the gingival sulcus, increases during treatment [13,14]. Studies also note a rise in bacterial species after treatment, such as Tannerella forsythia, primarily detected three months after the end of orthodontic therapy [15]. However, these studies generally analyze orthodontic therapy without specific validation of bacterial species during a particular treatment.

Focusing on the impact on bacterial flora, depending on the orthodontic appliances used, studies such as the longitudinal analysis conducted by Bhumika Shokeen et al. [16] suggest better oral health outcomes with clear appliances compared to traditional fixed appliances, correlated with oral microbial communities associated with plaque and gingival indices. However, these results contradict findings observed in the meta-analysis by Qian Wang et al. [17], which argues that the influence of Invisalign on the oral microbiome is not superior for oral health compared to fixed appliances. These contrasting conclusions require further investigation to determine the differences and microbiological alterations in oral cavities caused by these treatments. Regarding the ultimate goal of orthodontic appliances, which is to realign dental elements to achieve harmonized occlusion and spatial arrangement, the definitive superiority between the use of clear appliances and traditional fixed orthodontic therapy has not been established [18]. However, clear appliances are becoming increasingly appealing to patients for aesthetics and convenience as they are removable, thus enhancing comfort.

On the other hand, it is crucial to define their impact on the oral bacterial population as this is closely linked to the patient’s overall health. Therefore, this project aims to investigate, from both a microbiological perspective and in terms of oral and periodontal health indices, the two types of orthodontic appliances: traditional fixed metal braces and clear removable plastic appliances. The microbiological analysis aims to assess differences in bacterial species’ morphotypes. These results can be utilized to correlate any observed modifications with specific pathologies or clinical concerns [19,20,21]. A deeper understanding of the effects of a particular orthodontic approach will be crucial in applying more suitable procedures and considering the adoption of innovative technologies. This study hypothesizes that removable orthodontic appliances may lead to less plaque accumulation and better oral health indices; their removal by the user can allow for better oral hygiene since the mechanical load produced by these types of appliances is less constant than that exerted by fixed prostheses.

## 2. Materials and Methods

### 2.1. Sample Size

In this study, 60 patients (20 in fixed orthodontic therapy, 20 in clear aligner therapy, and 20 without any treatment) were enrolled, totaling 31 females and 29 males, aged between 12 and 65 years. The inclusion criteria were understanding and signing the informed consent form, being between 12 and 65 years old, as this is the age range where orthodontic appliances are most commonly used [22], and having proper oral hygiene. To standardize proper oral hygiene among enrolled patients, informational material outlining the recommended practices for personal oral hygiene was provided, which patients were required to adhere to. Exclusion factors from the study were considered to be subjects who were smokers, regular consumers of alcoholic beverages, patients with systemic diseases such as diabetes, HIV, and hepatitis, patients undergoing chemotherapy, pregnant women, and patients with caries, periodontal diseases, and/or dental implants. Patients were selected from the Department of Dentistry of Fondazione IRCCS Ca Granda Ospedale Policlinico in Milan upon invitation, provided they met the inclusion criteria The sample size was calculated using the software G*Power, version 3.1.9.6, to estimate the total number of cases required in a comparison study between different methods using repeated-measurement ANOVA tests. To obtain an experimental power of 80%, type I error probability of 0.05, and considering an effect size of 0.15, estimated based on a pilot study, for the Gram-positive bacteria quantity variables, the total sample size obtained is 69. The sphericity hypothesis has been considered. For the robustness of the data, a total of 75 patients will be considered in the experiment. After giving informed consent, the patients were divided into three groups: Group A (fixed orthodontic patients), Group B (clear aligner patients), and Group C (control group with no orthodontic treatment). Groups A and B were recruited from the Orthodontics Department, while the control group comprised patients attending dental check-ups from other departments (e.g., conservative dentistry). Demographic data for the study groups revealed the following: aligners group—age: 20, male: 11, female: 8, average number of teeth: 28; fixed group—age: 18, male: 9, female: 11, average number of teeth: 27; control group—age: 43, male: 9, female: 11, average number of teeth: 26.

Patients with periodontal disease diagnoses were excluded from the control group. All the participants underwent oral evaluations, including quantitative Full-Mouth Plaque Score (FMPS) and semi-qualitative (Silness and Löe Plaque Index) plaque indices, quantitative Full-Mouth Bleeding Score (FMBS) and semi-qualitative Modified Sulcus Bleeding index (mSBI), and periodontal health assessment (PSR). The one-year study aims to evaluate microbial flora differences in the three distinct cases.

### 2.2. Bleeding Indices

For the semi-qualitative assessment of periodontal inflammation, the Modified Mombelli Bleeding Index [23] (mSBI) was chosen, considering 6 surfaces per tooth (3 lingual/palatal and 3 vestibular) and assigning 4 different codes: Code 0 = absence of bleeding; Code 1 = bleeding upon probing with no redness or swelling; Code 2 = bleeding upon probing with redness and swelling; and Code 3 = spontaneous bleeding.

mSBI: Sum of codes assigned to individual periodontal sites divided by the number of analyzable sites with the maximum attributable code, and the whole ratio multiplied by 100.

Quantitative assessment was obtained through the O’Leary Full Mouth Bleeding Score (FMBS), considering six surfaces per tooth [24,25].

FMBS: Sum of individual periodontal sites with bleeding upon probing divided by the number of analyzed sites with the maximum attributable code, and the whole ratio multiplied by 100.

### 2.3. Plaque Indices

Using a plaque-disclosing tablet, two indices were recorded: for the semi-qualitative aspect, the Silness and Löe Plaque Index [26] was chosen, while for the quantitative aspect, the Full Mouth Plaque Score was used. Both indices divide the tooth into 6 surfaces: 3 lingual/palatal and 3 vestibular.

Biofilm assessment utilized a plaque-disclosing tablet, an explorer probe, a mirror, and an air and water syringe. Each dental surface was assigned a value from 0 to 3: Code 0 = dental surface without bacterial plaque; Code 1 = 1/3 of the crown covered with bacterial plaque; Code 2 = 2/3 of the crown covered with bacterial plaque; and Code 3 = >2/3 of the crown covered with bacterial plaque.

The Plaque Index (PI) is the sum of codes assigned to individual dental surfaces divided by the number of surfaces analyzed with the maximum attributable code, and the whole ratio multiplied by 100. Quantitative assessment was obtained through the FMPS, considering six surfaces per tooth.

FMPS: Sum of surfaces covered with plaque divided by the number of analyzed surfaces, and the whole ratio multiplied by 100.

PSR: An index used to evaluate the periodontal health of an individual patient. It precisely defines and addresses the needs of different patients, assessing the most suitable treatment, and identifying those requiring further, more accurate clinical investigations [27].

This examination divides dental arches into 6 sextants, proceeding with periodontal probing using a WHO periodontal probe. For each code, a different treatment type is identified: Codes 0 and 1 require evaluation and control of gingival biofilm, along with appropriate home oral hygiene instructions; Code 2 requires thorough tartar removal, reconstruction of overhanging restorations, and home oral hygiene instructions; and Codes 3 and 4 necessitate a comprehensive periodontal evaluation with an appropriate therapeutic program. The dental formula of the FDI Dental Numbering System was followed for this study.

### 2.4. Microbiological Analysis of Bacterial Plaque

Bacterial sub-gingival plaque analysis involved sampling from the lingual surface of tooth 3.1. Subgingival plaque samples were collected using a standardized procedure. After isolating and drying the area of interest, a periodontal probe was gently inserted into the gingival sulcus until reaching the base. Subsequently, lateral movement along the pocket’s base facilitated the dislodgement of subgingival plaque. Special care was taken to avoid tissue trauma during this process. It was decided to collect the plaque sample from the same tooth for all patients to standardize the collection. The lower incisor is also easily accessible, allowing for a simple and reproducible collection. All the samples were collected by the same dental hygienist, who also conducted the patient’s examination. The sample was placed in a sterile vial containing 0.5 mL of physiological saline and then sent to the laboratory.

The plaque samples were first disaggregated by agitating with quartz powder. After centrifugation, the supernatant containing bacteria was smeared onto five glass slides and fixed by heating the glass. Gram staining was performed to detect bacteria [26].

Once the coverslip was stabilized, a microscopic examination was performed at 1000× magnification using a 100× immersion objective. The microscope used was an Olympus CX43, with an Olympus LC30 camera and LCmicro Olympus software. For each patient, 3 slides were selected, and for each slide, 3 aggregates were chosen for analysis. 

The assessment involved both qualitative and semi-quantitative analyses. In the qualitative analysis, a detailed examination was carried out on the bacterial morphotypes identified within the aggregates. The predominant bacterial types analyzed included cocci, bacilli, spirochetes, yeasts/fungi, filamentous bacteria, and fusiform bacteria. For the semi-quantitative analysis, the parameters were set with 0 indicating the absence of a specific bacterial species, and 1 indicating its presence. The Gram staining technique also allowed for the differentiation of Gram-positive and Gram-negative bacteria, providing supplementary information in our reference table. We used 3 different individuals for the quantifications related to the microbiological characterizations. Then, an average of the values was calculated to ensure greater accuracy, as it is a sensitive operator procedure. For obtaining values related to the various periodontal indices, we adhered to the guidelines of the respective clinical practices.

### 2.5. Statistical Analysis

The collected data was analyzed using the Python programming language (Python Software Foundation. Python Language Reference, version 3.12. Available at http://www.python.org, accessed on 8 October 2023). The periodontal health statistical analysis was carried out considering the FMPS, PI, FMBS, mSBI, and PSR indices in the three groups, analyzing and comparing them. Normal assumptions of data distribution were evaluated using the Lilliefors test. The Kruskal–Wallis test was used to compare the three groups, and the Mann–Whitney test with Bonferroni correction was used for post hoc comparisons between two groups. The significance level was set to α = 0.05

## 3. Results

**Figure 1:**
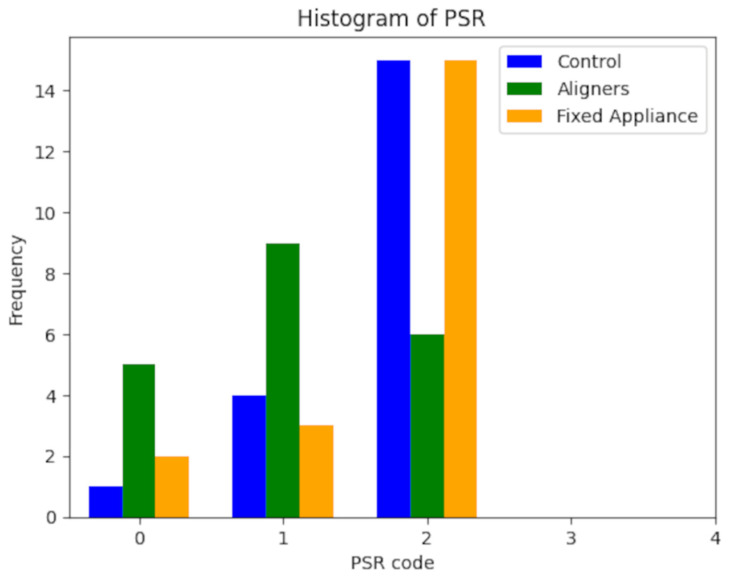
Histogram relative to the distribution of frequencies for PSR between the three groups.

The study involved 60 patients, evenly divided between men and women, aged between 12 and 65 years, with the number of dental elements ranging from 21 to 32. These patients were divided into three groups: controls, aligners, and fixed orthodontic therapy. To assess their periodontal health, we compared the FMPS, PI, FMBS, mSBI, and PSR indices in the three groups. Initially, a Lilliefors normality test was performed for the distributions related to the PI index in the three groups. Since these distributions were normal, and the sample size was relatively small (20 subjects per group), non-parametric statistical tests were chosen. Continuous variables were represented using the median and percentiles 25 and 75, while for the categorical variables (PSR), a bar chart was used to show frequencies in the three groups (Figure 1).

The three groups were compared using the Kruskal–Wallis test (Table 1). Post hoc comparisons between the two groups were conducted using the Mann–Whitney (Table 2) test with Bonferroni correction. The significance level was set at α = 0.05. The results in the descriptive statistics, including median, percentiles 25 and 75, and *p*-values derived from the Lilliefors test, are reported in Table 3. The PSR frequency is illustrated in Table 4. Results regarding the comparison between the three groups for the considered variables are presented in Table 4. There were significant differences (*p*-value < 0.05) in the FMPS, PI, and PSR measurements. The Mann–Whitney test with Bonferroni correction was applied to identify which groups differed from each other. This paired comparison revealed significant differences (*p*-corrected < 0.05), as indicated in the table.

The plaque collected from each patient was dissolved in the same volume of physiological solution (0.5 mL). Subsequently, the entire solution was placed on five slides, ensuring that at least one slide had a sufficient bacterial concentration for proper visualization under an optical microscope. This procedure was dictated by the inability to perform an accurate bacterial count at the time of collection, making it impossible to normalize all the samples. In more than one patient, bacteria were not detectable in the analyzed slides due to a low concentration and limitations associated with sample preparation. For this reason, one slide per patient was examined.

**Figure 2:**
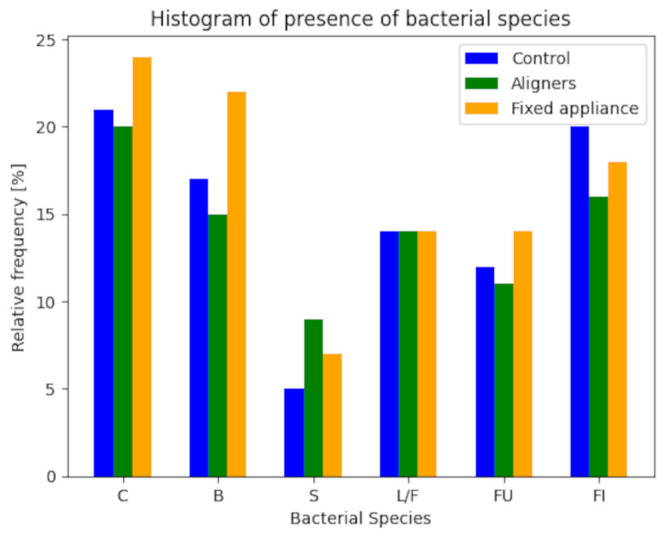
Relation between bacterial spaces and relative frequency.

**Figure 3:**
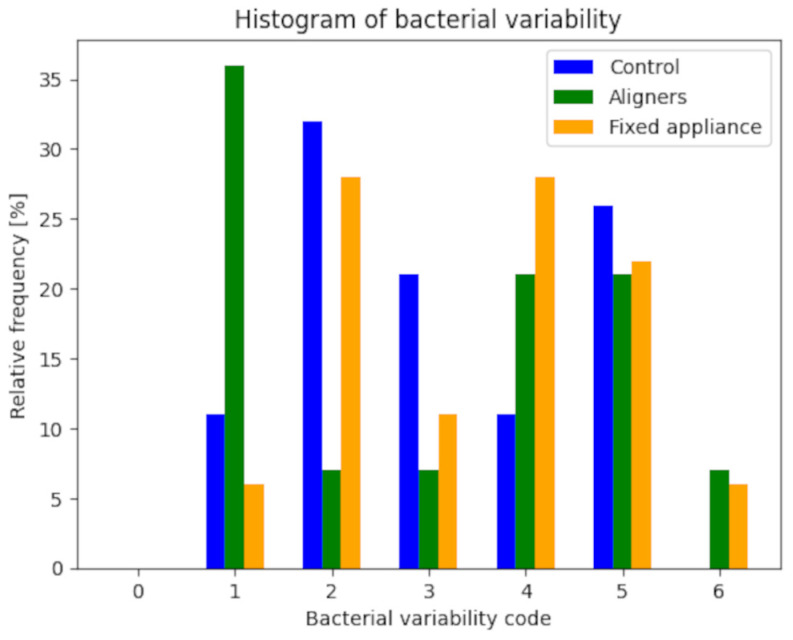
Bacteria variability.

For each bacterial species, the distributions of relative frequency were studied in the three groups (two experimental and one control), and the corresponding bar charts were created (Figure 2 and Figure 3). The analysis did not include patients for whom bacterial characterization data was unavailable. Therefore, the total sample for the control group consists of 19 patients, 14 patients in the aligners group, and 18 patients in the fixed appliances group. To account for the difference in group sizes, the tables report absolute and relative frequencies, while the bar charts represent relative frequencies.

The semi-quantitative analysis for microbiological characterization was based on assessing the presence or absence of five microbiological morphotypes. Each morphotype was represented by 0 in case of absence and 1 in case of presence. The bacterial variability index was obtained by summing the presence of different morphotypes (Table 5 and Table 6). This index was calculated for each of the three groups and subsequently analyzed using an appropriate statistical procedure, following the method used for the categorical variable PSR, as previously described.

The Lilliefors normality test yielded a significant result for all three groups (*p* < 0.05), indicating that the distributions do not follow a normal distribution. Therefore, the comparison between the three groups performed through the non-parametric Kruskal–Wallis test showed a non-significant result (*p* = 0.669), suggesting insufficient statistical evidence to claim significant differences between the three groups.

## 4. Discussion

The main objective of this study was to examine any relevant differences in oral and periodontal health parameters, as well as microbiological components, among patients undergoing two types of orthodontic treatments: clear aligners and traditional fixed orthodontic therapy. This analysis was conducted by comparing these two groups and a third control group, representing the average Italian patient and the general population—patients aware of the importance of proper oral hygiene, both professional and at home, who regularly undergo check-ups and tartar removal sessions at a frequency of approximately 6 months to 1 year. Regarding patients in orthodontic therapy, quarterly appointments are scheduled following the orthodontic protocol adopted at the Policlinico Hospital in Milan. The patients participating in the study are currently receiving treatment at this clinic, where they were recruited. Concerning the oral and periodontal health of the patients, it is known that the periodontal response to orthodontic appliances is influenced by various factors, including the general health of the host [28], systemic conditions’ presence, and dental plaque’s quantity and composition [29]. Additionally, lifestyle factors such as smoking can compromise periodontal support [30]. For these reasons, the study did not include smokers and patients with documented systemic conditions. Finally, oral hygiene practices play a crucial role in safeguarding periodontal health during orthodontic treatment. The presence of orthodontic appliances introduces modifications to the oral ecosystem, making the process of at-home oral hygiene more complex and promoting plaque accumulation around the attachments [31], hindering effective removal and creating retentive niches that facilitate bacterial colonization [32].

From the data analysis related to oral and periodontal health indices, statistically significant differences emerge, as confirmed by the conducted statistical analyses, between patients treated with aligners and those undergoing traditional fixed orthodontic therapy. The former shows a better condition in the oral and periodontal health indices, and this disparity can be attributed to various reasons. For example, Invisalign, thanks to the short intervals during which the device is not worn, such as during meals, contributes to reducing potential damage, decreasing the total amount of mechanical stress that the oral apparatus would otherwise be subjected to. In contrast, fixed orthodontic appliances impose constant and more significant traction on the periodontal ligament, increasing the likelihood of inflammation and bleeding—factors contributing to the deterioration in oral and periodontal health indices. Although the literature is not unanimous in reaching a conclusion on this matter, it is logical to think that a reduction, even if only sporadic, in mechanical stress, may positively affect overall oral health [33,34].

Moreover, from the perspective of oral hygiene, brackets pose a more significant obstacle to effective plaque removal than aligner attachments, favoring biofilm accumulation [35], retaining more plaque, and impeding its effective removal. This difficulty consequently leads to more pronounced inflammation, potentially creating a positive feedback effect as the mechanical action exerted by dental aligners causes chronic inflammation.

Regarding the microbiological aspect, the goal of this study was to verify if using one spacer over another would also result in a modification of the bacterial flora composition. We chose to perform a qualitative and semi-quantitative assessment of plaque to obtain a comprehensive and representative picture of the oral microflora in patients. The adopted approach was based on the analysis of bacterial micro-aggregates, where it was possible to ascertain the presence or absence of specific bacterial types based on their morphotype [36,37]. This procedure aimed to evaluate any macro-differences, as it was not sensitive; however, it proved easy and quickly executable. However, due to some critical issues in the used technique, we could not obtain statistically significant results. Therefore, to verify a potential difference in the composition of the bacterial flora, it was necessary to adopt a different method in plaque analysis, which could include genetic analysis to obtain more informative results [38].

This study was initiated with the goal of analyzing the various uncertainties concerning the differentiations that distinguish two different orthodontic treatment modalities: treatment with clear aligners and traditional fixed orthodontic therapy. The aspects considered worthy of further investigation include, on the one hand, the microbiological aspect and, on the other hand, the patient’s oral and periodontal health conditions, evaluated through the respective indices. Regarding the latter aspect, significant results were achieved, accompanied by several plausible explanations. The results demonstrate that patients undergoing treatment with clear aligners have better periodontal conditions. This phenomenon can be explained by clear aligners being easily removed during meals and oral hygiene procedures. This allows patients to maintain effective control of gingival biofilm and, consequently, to preserve relatively healthy periodontal conditions during orthodontic treatment. It differs from traditional fixed appliances, where elements used in therapy, such as bands and archwires, promote biofilm accumulation, retain more plaque, and hinder effective removal. Biofilm removal is a fundamental aspect, as the higher the accumulation of bacterial plaque, the greater the likelihood of developing an inflammatory process due to bacterial microbiota proliferation [39]. Dental plaque is the principal etiological agent in the development of gingivitis and, consequently, plays a crucial role in the possible evolution and progression towards periodontitis [40]. Another plausible explanation for the difference between the two oral and periodontal health indicators could be related to less mechanical stress exerted by aligner orthodontic appliances on the periodontium [41,42,43]. This could be attributed to the fact that they do not apply constant pressure, as they can be easily removed at certain times of the day. In contrast, treatment with traditional fixed orthodontics involves constant tension on the periodontal ligament, which could explain the increase in inflammation and bleeding and, consequently, a lower index of oral and periodontal health. This hypothesis could be a stimulating point of interest for further analysis and investigation. Lastly, it was not feasible to consider the potential influence of aligner materials on oral flora for evaluation and comparison purposes. While fixed orthodontic appliances were uniformly made of medical-grade steel, the exact material composition of the removable aligners is unknown due to patent reasons. However, they are known to be made of plastic material. Nonetheless, it would be beneficial to delve deeper into this aspect, as the use of specific materials could significantly impact oral bacterial load, thus potentially enhancing patient treatment [44].

The limitations of this study are related to the patient’s protocol for home oral hygiene. It is evident that it is difficult to be certain about the attention each patient pays to home oral hygiene, and consequently, this impacts the overall outcome. Further studies investigating the clinical relevance of the differences between the impact of clear aligners and fixed appliances on periodontal health status are needed for definitive conclusions. Another limitation was the broad age range, which could impact the comparison of oral microflora composition. On the other hand, it is essential to consider that the factors influencing plaque composition are numerous, making it extremely difficult to find a sufficiently large and homogeneous group that would still be representative of a small segment of the population or a particular subset [45].

## 5. Conclusions

The study provides initial insights into the impact of two dental aligners on oral health. With an expanded sample size, we anticipate more precise distinctions between their effects. Additionally, enhancing the analysis of resident bacteria could yield more conclusive findings. There were significant differences (*p*-value < 0.05) in the FMPS, PI, and PSR measurements. Further investigation into the individual effects of each orthodontic appliance on periodontal health is warranted due to variations in patients’ home oral hygiene practices.

## Figures and Tables

**Table 1:** Results of Kruskal–Wallis test between groups. * statistical significance.

	FMPS	PI	FMBS	mSBI	PSR
*p*-value	0.006 *	0.000 *	0.860	0.712	0.010 *

**Table 2:** Results of Mann–Whitney test between groups. * statistical significance.

Group Comparison	FMPS	PI	PSR
Aligners–Control	0.006 *	0.001 *	0.029 *
Aligner–Fixed Appliance	0.356	0.686	0.045 *
Fixed Appliance–Control	0.159	0.002 *	2.630

**Table 3:** Descriptive statistics of qualitative variables: 25th percentile, median, 75th percentile, and *p*-value relative to normality test. * statistical significance.

	Control				Aligners				Fixed Appliance			
	FMPS	PI	FMBS	mSBI	FMPS	PI	FMBS	mSBI	FMPS	PI	FMBS	mSBI
25% perc	100	72	3	1	76	37	0	0	91	53	0	0
Median	100	84	9	3	93	55	8	3	100	64	7	3
75% perc	100	92	23	10	100	69	18	6	100	73	23	14
Lilliefors *p*-value	0.001 *	0.201	0.046 *	0.005 *	0.002 *	0.834	0.017 *	0.011 *	0.001 *	0.564	0.001 *	0.001 *

**Table 4:** Distribution of frequencies for PSR between the three groups: control, aligners, and fixed appliances.

PSR			
Code	Control	Aligners	Fixed A.
0	1	5	2
1	4	9	3
2	15	6	15
3	0	0	0

**Table 5:** Semi-quantitative evaluation of the amount of bacteria detected upon morphotype.

	Control		Aligners		Fixed Appliance	
Bacterial Species	Absolute	Relative [%]	Absolute	Relative [%]	Absolute	Relative [%]
C	16	21	13	20	18	24
B	12	17	8	15	16	22
S	0	5.3	2	9.1	1	6.6
L/F	9	14	7	14	8	14
FU	7	12	4	11	8	14
FI	15	20	9	16	12	18

**Table 6:** Values of the bacteria frequency.

	**Control**		**Aligners**		**Fixed Appliance**	
**Code**	**Absolute**	**Relative [%]**	**Absolute**	**Relative [%]**	**Absolute**	**Relative [%]**
0	0	0	0	0	0	0
1	2	11	5	36	1	6
2	6	32	1	7	5	28
3	4	21	1	7	2	11
4	2	11	3	21	5	28
5	5	26	3	21	4	22
6	0	0	1	7	1	6
tot	19	100	14	100	18	100
	**Control**		**Aligners**		**Fixed Appliance**	
**Code**	**Absolute**	**Relative [%]**	**Absolute**	**Relative [%]**	**Absolute**	**Relative [%]**
0	0	0	0	0	0	0
1	2	11	5	36	1	6
2	6	32	1	7	5	28
3	4	21	1	7	2	11
4	2	11	3	21	5	28
5	5	26	3	21	4	22
6	0	0	1	7	1	6
tot	19	100	14	100	18	100

## Data Availability

The data presented in this study are available on reasonable request, after the signature of a formal data sharing agreement in anonymous form, from the corresponding author because they are protected by privacy.
